# Enhanced Antileukemic Efficacy of Venetoclax-Loaded PEG-PLGA Nanoparticles Against THP-1 Acute Monocytic Leukemia Cells via Suppression of BCL-2 and Pro-Survival Signaling Pathways

**DOI:** 10.3390/biomedicines14102259

**Published:** 2026-10-06

**Authors:** Nermin Keni Begendi, Elif Kaga

**Affiliations:** 1 Division of Hematology, Department of Internal Medicine, School of Medicine, Afyon Health Sciences University, Afyonkarahisar 03030, Türkiye; 2 Department of Biochemistry, Faculty of Pharmacy, Afyon Health Sciences University, Afyonkarahisar 03030, Türkiye; elif.kaga@afsu.edu.tr

**Keywords:** venetoclax, PEG-PLGA nanoparticles, acute myeloid leukemia, BCL-2 inhibition, apoptosis, ERK1/2 signaling, NF-κB pathway, nanomedicine, drug delivery

## Abstract

**Background:** Acute myeloid leukemia (AML) presents a significant therapeutic challenge due to the systemic toxicity associated with traditional chemotherapy. Venetoclax, a selective BCL-2 inhibitor, has shown promising antileukemic activity; however, limited pharmacokinetic properties impair its clinical use. Polymeric nanocarriers composed of PEG-PLGA provide a biocompatible delivery system that enhances drug stability, controls drug release kinetics, and increases intracellular drug concentration. **Methods:** The physicochemical characteristics of VEN-PEG-PLGA nanoparticles were assessed using dynamic light scattering (DLS), scanning electron microscopy (SEM), drug loading analysis, and in vitro drug release studies. The antileukemic potential was evaluated in THP-1 cells using the CCK-8 viability assay, cell cycle profiling, Annexin V-FITC/PI apoptosis quantification, and Western blot analysis of Bcl-2, cleaved caspase-3, cleaved caspase-9, ERK1/2, phospho-ERK1/2 (p-ERK1/2), NF-kB, and phospho-NF-kB (p-NF-kB). **Results:** The VEN-PEG-PLGA nanoparticles displayed an average particle size of 186 ± 4 nm, polydispersity index of 0.195 ± 0.005 with loading efficiency of 89%, and loading capacity of 6.26%. These nanoparticles exhibited a pH-sensitive drug release profile, with approximately 72–74% cumulative release at pH 7.4 and 88–89% at pH 5.5 after 96 h. In THP-1 cells. Both free venetoclax and VEN-PEG-PLGA nanoparticles exhibited concentration-dependent cytotoxicity, although free venetoclax was more cytotoxic with EC50 values of 5.59 × 10^−7^ M (95% CI: 1.27 × 10^−7^–2.46 × 10^−6^ M) and 1.35 × 10^−6^ M (95% CI: 2.64 × 10^−7^–6.96 × 10^−6^ M) for the free drug and nanoparticle form, respectively. Cell cycle analysis indicated an increase in the G0/G1 cell population in the control group following treatment with VEN-PEG-PLGA nanoparticles, along with a reduction in the G2/M phase population. Annexin V-FITC/PI analysis demonstrated that VEN-PEG-PLGA nanoparticles increased the ratio of late apoptotic cells, compared with free venetoclax treatment. Western blot analysis showed decreased expression of Bcl-2, p-ERK1/2, and p-NF-kB, along with increased levels of cleaved caspase-3 and cleaved caspase-9 following treatment, with more pronounced changes observed in the group treated with VEN-loaded PEG-PLGA nanoparticles. **Conclusions:** VEN-PEG-PLGA nanoparticles modulate BCL-2/ERK1/2/NF-κB-related apoptotic responses, cell cycle kinetics, and signaling pathways in THP-1 AML cells, providing in vitro evidence supporting preclinical research on this nanoformulation in the context of AML.

## 1. Introduction

AML is a malignant disease, characterized by the uncontrolled proliferation of immature myeloid progenitor cells, which disrupts the normal structure of the bone marrow [1,2]. Despite recent advances in treatment, achieving sustained remission remains a challenging goal particularly among elderly patients and those with adverse cytogenetics [3,4]. The lack of selectivity, off-target organ damage, and tendency toward clonal resistance associated with traditional induction regimens underscore the urgent need for molecularly targeted therapies [5].

Among the molecular markers of AML, increased BCL-2 expression is particularly notable because of its association with chemotherapy resistance and poor prognosis [1,2,3,4]. BCL-2 preserves mitochondrial membrane integrity by binding to BAX and BAK, thereby inhibiting cytochrome c release and consequently caspase activation [6]. Venetoclax restores sensitivity to apoptosis by binding to the BCL-2 protein and exerts a significant antileukemic effect, particularly when used in combination with hypomethylation agents [7,8].

In addition to BCL-2, various survival-promoting signaling pathways play roles in AML pathogenesis and resistance to treatment. The ERK1/2 kinase cascade maintains the proliferation tendency of cells in AML and reduces sensitivity to cytotoxic therapy [9]. Similarly, constitutive NF-κB signaling supports the survival of leukemic cells by regulating anti-apoptotic gene targets [1]. PLGA- and PEG-PLGA-based polymeric nanocarrier platforms are attracting growing interest in the field of nanomedicine as they can overcome pharmacokinetic barriers, enhance the water solubility and facilitate the drug’s selective delivery into cells [10,11,12,13,14].

Derived from an acute monocytic leukemia patient, the THP-1 cell line serves as a well-validated in vitro platform for investigating monocyte–macrophage physiology and the antileukemic potential of target compounds [14]. Its documented sensitivity to BCL-2 inhibition renders it a suitable model for evaluating venetoclax-based therapies.

The objectives of this study are as follows: (i) to synthesize VEN-loaded PEG-PLGA nanoparticles for the treatment of AML and to determine their physicochemical properties; (ii) to investigate pH-dependent drug release in acidic intracellular endosomal and lysosomal compartments; (iii) to evaluate their antileukemic activity in THP-1 acute monocyte leukemia cells; and (iv) to elucidate the mechanistic basis of the nanoformulation’s effects on apoptosis and signal transduction via the BCL-2, ERK1/2, and NF-κB pathways.

## 2. Materials and Methods

### 2.1. Materials

Poly (ethylene glycol) methyl ether-block-poly(lactide-co-glycolide) (PEG Mn 5000, PLGA Mn 7000) and venetoclax (purity ≥ 99%) were obtained from Sigma-Aldrich. Acetone (HPLC), dimethyl sulfoxide (DMSO), and other reagents were obtained from Merck. RPMI-1640 and FBS were obtained from Biowest (Nuaillé, France). The antibodies used were NF-κB p65 (clone 572) and phospho-NF-κB p65 (Ser337) from Invitrogen; ERK1/2 and phospho-ERK1/2 (Thr202/Tyr204; clone MILAN8R, Invitrogen/eBioscience Waltham, MA, USA); caspase-3 (ab44976) and caspase-9 (ab52298) from Abcam (Cambridge, United Kingdom); and β-actin (sc-47778, Santa Cruz Biotechnology, Dallas, TX, USA ).

### 2.2. Preparation of VEN-PEG-PLGA Nanoparticles

VEN-PEG-PLGA NPs were fabricated using an emulsion-solvent evaporation procedure. PEG-PLGA (2 mg) and venetoclax (0.15 mg) were co-dissolved in 500 µL acetone and combined with 500 µL distilled water under stirring. The resulting emulsion was maintained on an orbital shaker overnight at ambient temperature to allow complete acetone removal from the system. The unentrapped drug was separated by centrifugation at 6000 rpm, and the nanoparticle-containing supernatant was collected. The pellet was reconstituted in DMSO and spectrophotometrically quantified using a calibration curve to determine the amount of unencapsulated venetoclax. Blank PEG-PLGA nanoparticles were prepared without venetoclax.

The amounts of encapsulated drug (EE%) and drug loading (DL%) were determined using the following equations:

EE% = (mass of drug in nanoparticles/total drug added) × 100

DL% = (mass of drug in nanoparticles/total nanoparticle mass) × 100

### 2.3. Physicochemical Characterization

The hydrodynamic size and polydispersity index (PDI) of the PEG-PLGA nanoparticles were assessed using dynamic light scattering (DLS) (Zetasizer Nano ZS, Malvern Instruments, Malvern, UK) in triplicate. Surface morphology was characterized by SEM (SEM; Zeiss LEO 1430 VP, Oberkochen, Germany) after sputter-coating the samples with gold (Bal-Tec, Balzers, Switzerland).

### 2.4. In Vitro Drug Release Study

In vitro drug release from VEN-PEG-PLGA nanoparticles was evaluated under two different pH conditions, using PBS (pH 7.4) and acetate buffer (pH 5.5) as the release media to mimic physiological and mildly acidic intracellular environments, respectively. A nanoparticle suspension volume was mixed with 1.5 mL of each release medium and maintained at 37 °C. Samples collected at 0, 24, 48, and 96 h were measured spectrophotometrically at 286 nm using a calibration curve for quantification.

### 2.5. Cytotoxicity Assay

THP-1 leukemia cells were maintained in RPMI-1640 as the basal medium, containing 10% fetal bovine serum (FBS) and penicillin–streptomycin (100 U/mL), at 37 °C in a humidified atmosphere with 5% CO_2_. Cells were seeded into 96-well plates (48-h treatment period applied following seeding at a density of 3 × 10^3^ cells per well) with free VEN or VEN-PEG-PLGA nanoparticles at concentrations of 10^−12^–10^−3^ M. Cell viability was assessed by the Cell Counting Kit-8 (CCK-8) assay (Sigma-Aldrich, St. Louis, MO, USA). CCK-8 solution (10% *v*/*v*) was added to each well, and the cells were incubated for 4 h at 37 °C. The absorbance was subsequently recorded at 450 nm using a Multiskan SkyHigh microplate reader (Thermo Fisher Scientific, Waltham, MA, USA). Viability values were calculated relative to the untreated control group and expressed as percentages (*n* = 3). EC50 values were determined using GraphPad Prism software (version 8; GraphPad Software, San Diego, CA, USA).

### 2.6. Cell Cycle Analysis

THP-1 cells were treated with free venetoclax (VEN) or VEN encapsulated in PEG-PLGA nanoparticles (VEN-PEG-PLGA NPs), both at a concentration of 5.59 × 10^−7^ M, for 48 h. THP-1 cells were fixed with 70% ethanol and washed with phosphate-buffered saline. Following fixation, the cells were incubated with a propidium iodide (PI) staining solution containing RNase A for 10 min in the dark. Cell cycle distribution was assessed by measuring DNA content with a DxFLEX flow cytometer (Beckman Coulter, Brea, CA, USA).

### 2.7. Apoptosis Analysis

Apoptotic cell death was assessed with an Annexin V-FITC Apoptosis Kit (Beckman Coulter, Brea, CA, USA). following the kit protocol. THP-1 cells (5 × 10^4^ per well) were exposed to free VEN or VEN-PEG-PLGA NPs at a concentration of 5.59 × 10^−7^ M for 48 h. The treated THP-1 cell suspensions were centrifuged at 200× *g* for 5 min. The cells were then simultaneously stained with Annexin V-FITC and propidium iodide (PI) in the dark at room temperature for 15 min. Early and late apoptotic cell populations were subsequently quantified by flow cytometry using a DxFLEX system (Beckman Coulter, Brea, CA, USA).

### 2.8. Western Blot Analysis

Protein concentrations in the cell lysates were determined using a BCA assay, with BSA as the reference standard. Equal amounts of protein (10 µg per lane) were separated by 12% SDS-PAGE and subsequently transferred onto nitrocellulose membranes for analysis of the proteins of interest. The nitrocellulose membranes were first blocked with 5% skim milk prepared in TBS-T for 1 h at room temperature. The membranes were subsequently incubated overnight at 4 °C with the appropriate primary antibodies. After three washes with TBS-T, HRP-conjugated secondary antibodies were applied for 1 h at room temperature. The immunoreactive bands were detected using an enhanced chemiluminescence (ECL) substrate and imaged and analyzed using a Thermo Scientific iBright imaging system.

### 2.9. Statistical Analysis

Data were shown as mean ± standard deviation (SD) from three independent experiments (*n* = 3). Statistical analyses were performed using GraphPad Prism (version 8.0, GraphPad Software, San Diego, CA, USA). To assess differences among the experimental groups, a two-way analysis of variance (ANOVA) was used, followed by Tukey’s multiple-comparison test. We considered *p* < 0.05 statistically significant and *p* < 0.01 highly significant.

### 2.10. Ethical Statement

As a commercially available cell line (THP-1) was used in this study, and no human or animal subjects were involved, institutional review board approval was not required.

## 3. Results

### 3.1. Physicochemical Characterization of VEN-PEG-PLGA NPs

The physicochemical properties of the PEG-PLGA nanoparticles were evaluated using dynamic light scattering (DLS) and scanning electron microscopy (SEM). DLS analysis revealed a mean hydrodynamic diameter of 186 ± 4 nm and a polydispersity index (PDI) of 0.195 ± 0.005, indicating a narrow and homogeneous particle size distribution (Figure 1A). SEM confirmed the spherical nanoparticle morphology, which was consistent with the DLS particle size distribution (Figure 1B).

### 3.2. Drug Loading Efficiency and Capacity

The performance of the VEN-loaded PEG-PLGA nanoparticles in terms of drug loading was assessed by examining the loading efficiency (LE) and loading capacity (LC). The loading efficiency was 89%, indicating that approximately 0.13 mg of the original 0.15 mg of venetoclax was effectively encapsulated within the nanoparticles. The loading capacity was measured at 6.26%.

### 3.3. In Vitro Drug Release

VEN-PEG-PLGA NPs exhibited a pH-responsive drug release profile (Figure 2). At physiological pH 7.4, cumulative drug release increased gradually over time, reaching approximately 72–74% after 96 h, thus indicating sustained release. In contrast, drug release was accelerated under acidic conditions (pH 5.5), with approximately 58–60% of the encapsulated venetoclax released within the first 48 h and a cumulative release of approximately 88–89% after 96 h of incubation.

### 3.4. Cytotoxicity Assessment

The CCK-8 assay demonstrated the concentration-dependent cytotoxicity of both free venetoclax (VEN) and VEN-PEG-PLGA nanoparticles (VEN-PEG-PLGA NPs) in THP-1 cells after 48 h of treatment (Figure 3A). Free VEN exhibited greater cytotoxicity than the nanoparticle formulation, with EC50 values of 5.59 × 10^−7^ M and 1.35 × 10^−6^ M, respectively (Figure 3B).

### 3.5. Cell Cycle Analysis Results

Flow cytometry was used for cell cycle analysis to assess the impact of free venetoclax (VEN) and VEN-PEG-PLGA nanoparticles on THP-1 cell cycle progression (Figure 4). In the control group, cells were distributed across the G0/G1, S, and G2/M phases at 49.30%, 21.77%, and 28.80%, respectively. With free VEN, the percentage of cells in G0/G1 increased to 57.52%, whereas that in G2/M decreased to 22.62%. A similar trend was observed with VEN-PEG-PLGA treatment, where 61.04% of cells were in G0/G1 and 18.68% in G2/M, with the S phase remaining similar to the control group (19.33%). Compared to free VEN, VEN-PEG-PLGA resulted in a greater percentage of G0/G1 (61.04% vs. 57.52%) and a smaller percentage of G2/M (18.68% vs. 22.62%), whereas the S-phase populations were nearly identical (19.33% vs. 19.27%). A one-way ANOVA, combined with Tukey’s multiple comparison test, revealed that there was no statistically significant difference among the groups (*p* > 0.05).

### 3.6. Apoptosis by Annexin V/PI Flow Cytometry

Annexin V-FITC/PI staining was used to assess apoptosis induction by free venetoclax (VEN) and VEN-PEG-PLGA nanoparticles. The control group exhibited a high level of cell viability, with 96.16% of cells remaining viable. When treated with free VEN, early apoptotic cells increased to 16.99% and late apoptotic cells to 22.13%, compared to the control group. VEN-PEG-PLGA nanoparticle treatment led to an increase in late apoptosis to 36.99%, whereas the number of viable cells decreased to 51.52% (Figure 5A). The analyses confirmed that both free VEN and the nanoparticle formulation increased apoptosis compared to the control group, with the nanoparticles resulting in a greater increase in late-stage apoptosis (*p* < 0.05) (Figure 5B).

### 3.7. Western Blot Analysis of Signaling Proteins

Western blotting was used to assess the levels of apoptosis- and survival-related proteins in THP-1 cells following treatment with free venetoclax (VEN), blank PEG-PLGA nanoparticles, or VEN-PEG-PLGA nanoparticles (Figure 6A). Bcl-2 protein expression was decreased in the treatment groups, with a significant reduction in the VEN-PEG-PLGA NP group compared with the control group (*p* < 0.05) (Figure 6B). In contrast, cleaved caspase-3 and cleaved caspase-9 protein expression increased after treatment, with higher levels observed in the VEN-PEG-PLGA NP group compared with the free VEN group.

Analysis of cell survival-associated proteins revealed reduced ERK1/2 and p-ERK1/2 expression in the treatment groups compared to the control group. Similarly, p-NF-κB level was reduced after treatment, with a significant decrease in the VEN-PEG-PLGA group compared to that in the control (*p* < 0.05). Total NF-κB expression showed minor changes between the experimental groups. Overall, VEN-PEG-PLGA treatment reduced Bcl-2, ERK1/2, p-ERK1/2, and p-NF-κB levels and increased cleaved caspase-3 and cleaved caspase-9 levels, indicating the modulation of apoptosis- and survival-related signaling in THP-1 cells (Figure 6B).

## 4. Discussion

This study describes the fabrication and biological characterization of VEN-PEG-PLGA nanoparticles as a nanomedicine approach for the management of AML. PEG-PLGA nanoparticles were engineered as biodegradable carriers designed to promote intracellular drug delivery while modulating apoptosis-associated molecular networks. Compared to the free drug, the nanoformulation exerted more pronounced effects on apoptotic process, cell cycle regulation, and survival-related signal transduction.

The VEN-PEG-PLGA nanoparticles exhibited a hydrodynamic diameter of 186 ± 4 nm and a PDI value of 0.195 ± 0.005, consistent with a well-defined monodisperse preparation. In THP-1 cell viability assays, both formulations caused dose-dependent cytotoxicity; the lower EC50 value for free venetoclax (5.59 × 10^−7^ M versus 1.35 × 10^−6^ M for the nanoparticle form) indicated that encapsulation reduced acute in vitro activity of venetoclax. At 186 nm, these nanoparticles fall within the nanoscale range for polymeric drug delivery, potentially enhancing cellular interactions and biological transport [10,11].

Surface PEGylation confers a net negative charge that limits serum opsonin adsorption and mononuclear phagocyte recognition, thereby prolonging systemic circulation and creating conditions favorable for passive tumor accumulation [11,15]. The 89% encapsulation efficiency confirms that venetoclax is effectively trapped within the polymer matrix, while the low PDI value of 0.195 indicates a high degree of size uniformity [16,17]. The pH-responsive release behavior of the nanoparticles is mechanistically relevant: after endocytic uptake, nanoparticles transit through compartments with pH values between 4.5 and 5.5, which accelerates PLGA matrix degradation in the cytoplasm [15,18]. The substantially higher release fraction at pH 5.5 (88%) relative to that at pH 7.4 (72%) is consistent with acid-triggered drug liberation following cellular internalization [18].

After 48h, free venetoclax exhibited higher acute cytotoxicity than VEN-PEG-PLGA nanoparticles, as evidenced by its lower EC50 value. In contrast, VEN-PEG-PLGA nanoparticles had stronger effects on apoptosis, cell cycle regulation, and the expression of apoptosis- and survival-related proteins. This lower EC50 suggests that nanoparticle encapsulation does not increase cytotoxic potency in this model but modifies responses through apoptosis, cell cycle, and survival-related signaling pathways [10,11].

Compared to free VEN, the nanoparticle formulation resulted in greater cell accumulation in the G0/G1 compartment (57.52% vs. 61.04%) and, consequently, a decrease in the G2/M fraction (22.62% vs. 18.68%) (*p* > 0.05). In the apoptosis analysis, the VEN-PEG-PLGA nanoparticles led to a greater increase in late-stage apoptosis (*p* < 0.05). The prolonged intracellular retention of venetoclax, released from PEG-PLGA matrices, may allow for the maintenance of concentrations that activate control points, beyond those achieved with free drug [18].

The MOMP-mediated caspase signaling pathway constitutes the canonical effector pathway of intrinsic apoptosis regulated by the BCL-2 family [19]. Protein expression profiling by Western blot revealed that VEN-PEG-PLGA nanoparticles significantly reduced levels of Bcl-2, total and phosphorylated ERK1/2, and phospho-NF-κB in THP-1 cells, while simultaneously increasing levels of cleaved caspase-3 and caspase-9 (*p* < 0.05). Increased caspase activation with VEN-PEG-PLGA nanoparticles may reflect sustained intracellular release, maintaining venetoclax concentrations that are sufficient to counter residual anti-apoptotic signals [20]. Prolonged ERK1/2 activation drives venetoclax resistance in AML by inducing MCL-1 and related survival proteins [9,21,22]. Concurrent inhibition of the NF-κB signaling pathway eliminates a secondary survival pathway by suppressing the transcription of MCL-1 and BCL-XL, thereby rendering cells susceptible to apoptosis induced by BCL-2 inhibitors [23,24].

The mechanism linking nanoparticle delivery to enhanced ERK1/2 and NF-κB inhibition may involve sustained intracellular release of venetoclax [18]. Intracellular PLGA hydrolysis produces lactic and glycolic acid degradation products, creating an acidic microenvironment that promotes pH-triggered release [18]. However, distinguishing these contributions requires investigation using inhibitors and pH monitoring.

The nanoformulation enhances apoptotic signaling pathways rather than cytotoxicity, compared to free venetoclax.

Several limitations must be considered. First, the isolated use of THP-1 cells, a monocyte-derived subtype, limits the generalizability of the findings across the heterogeneous AML phenotype; confirmatory experiments must be conducted in alternative cell lines as well as in patient-derived blasts. Second, the absence of a blank nanoparticle control in the Annexin V/PI experiment means that a direct carrier-attributed contribution to the apoptotic signal cannot formally be excluded. Third, in vivo pharmacokinetic profiling and efficacy testing in mouse AML models are the prerequisites for developing this formulation. Fourth, lyophilization stability under standard storage conditions and behavior in complex biological matrices, such as whole blood and bone marrow aspirates, require systematic evaluation.

From a translational perspective, the proven clinical safety of FDA-approved PLGA-based products (e.g., Lupron Depot and Risperdal Consta) provides a regulatory framework that could facilitate new drug applications for research purposes. Future studies should include active targeting compounds while preserving CD34+ normal hematopoietic progenitor cells—an effective next step toward improving venetoclax’s therapeutic index.

## 5. Conclusions

This study demonstrates that VEN-PEG-PLGA nanoparticles are a promising nanomedicine strategy for AML treatment. The nanoformulation exhibited physicochemical properties, including nanoscale particle size, high encapsulation efficiency, narrow size distribution, and pH-responsive drug release profile, and VEN-PEG-PLGA nanoparticles activated the intrinsic caspase apoptotic pathway and modulated apoptosis- and survival-related proteins. These findings support PEG-PLGA nanoparticle encapsulation to enhance the effects of venetoclax in AML treatment.

However, to confirm the therapeutic efficacy of this nanoformulation, further in vivo pharmacokinetic and efficacy studies and validation studies in additional AML cell models and primary patient samples are required.

## Figures and Tables

**Figure 1:**
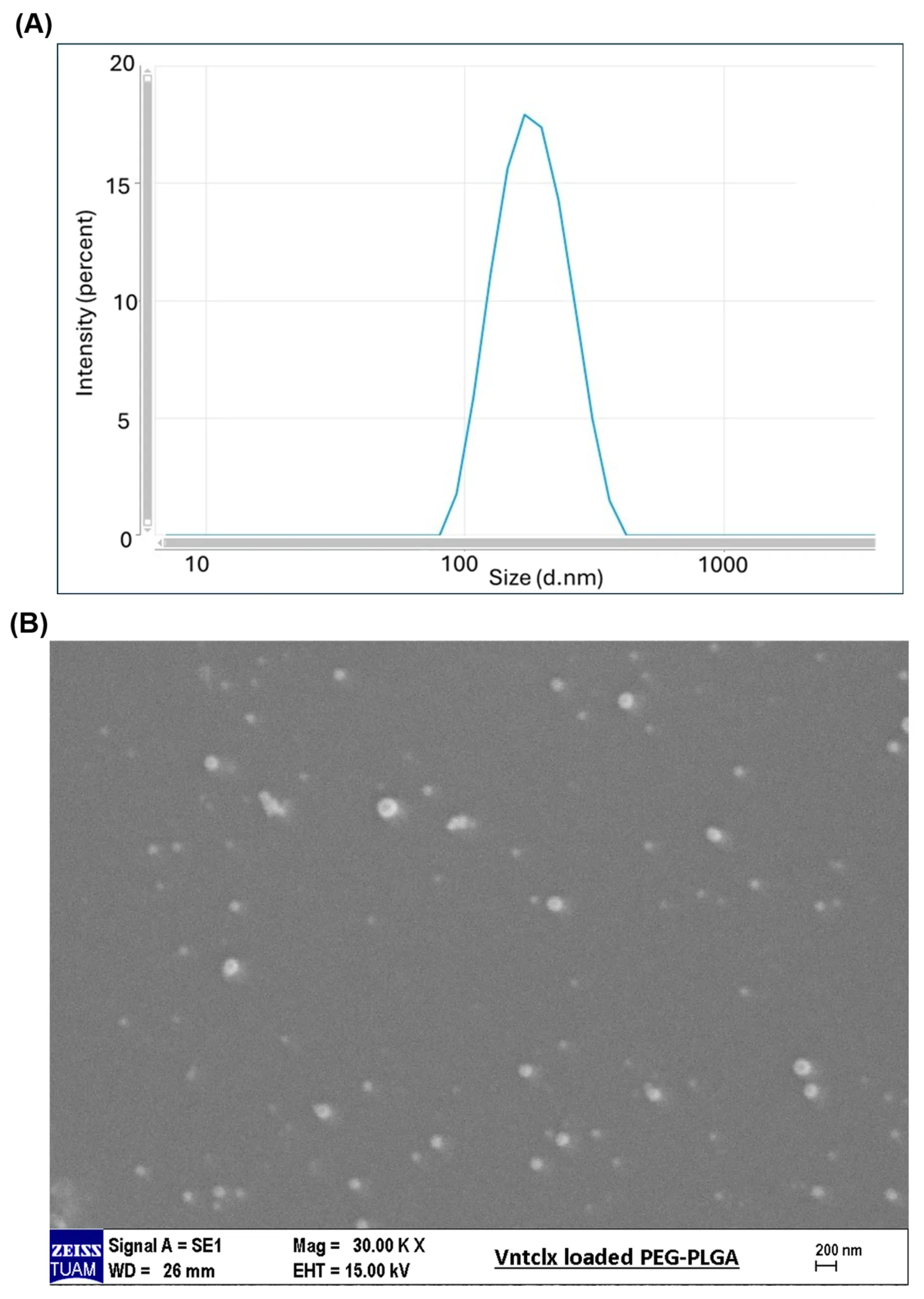
(**A**) Particle size distribution of VEN-PEG-PLGA NPs measured using dynamic light scattering (DLS). (**B**) SEM image of the VEN-PEG-PLGA nanoparticles exhibiting predominantly spherical morphology. Scale bar: 200 nm.

**Figure 2:**
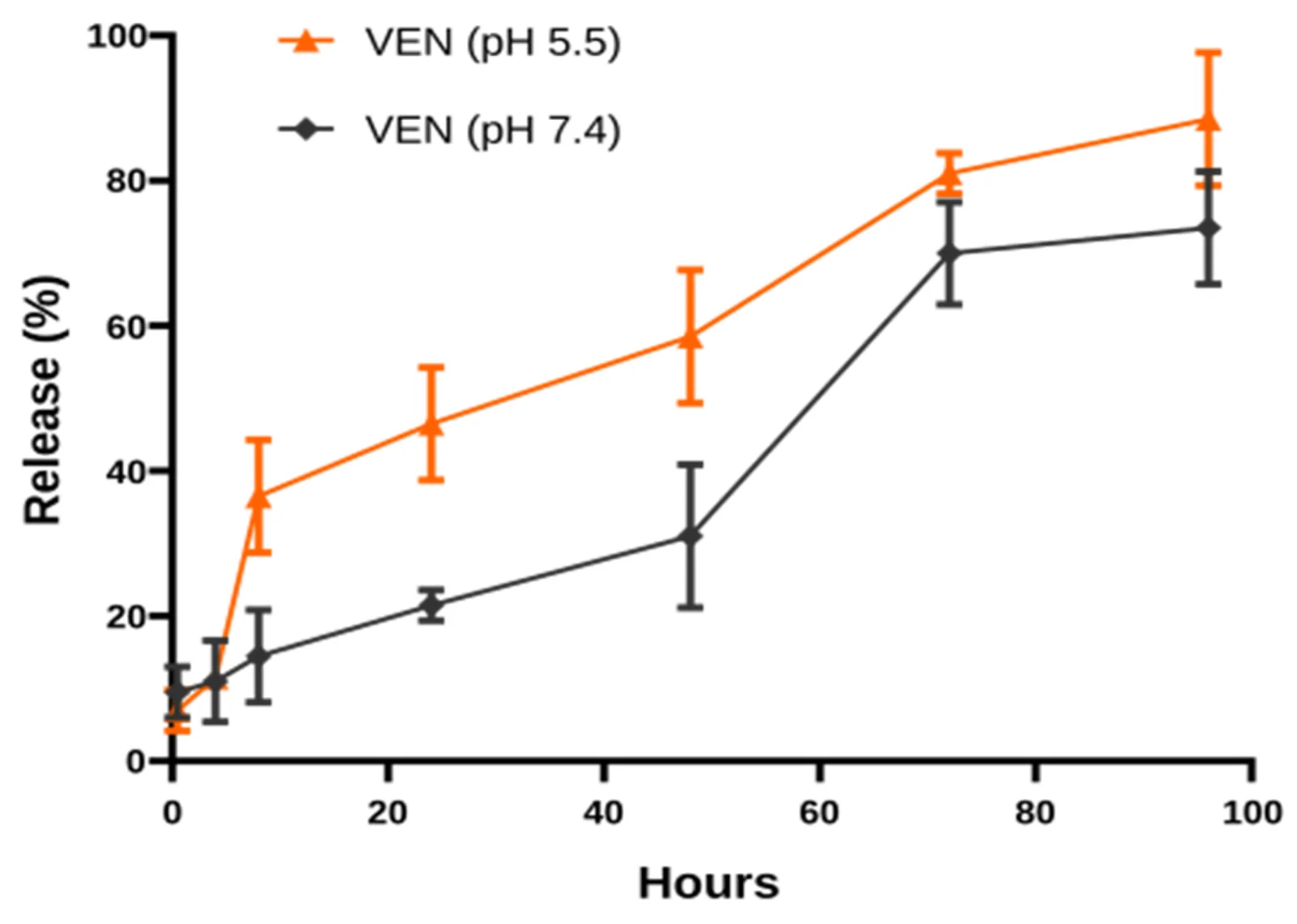
In vitro release profile of venetoclax-loaded PEG-PLGA nanoparticles (VEN-PEG-PLGA NPs) at pH 7.4 and 5.5 over 96 h. Drug release studies were performed at 37 °C, and the cumulative drug release (%) was determined at predetermined time intervals. Data are presented as mean ± SD (*n* = 3).

**Figure 3:**
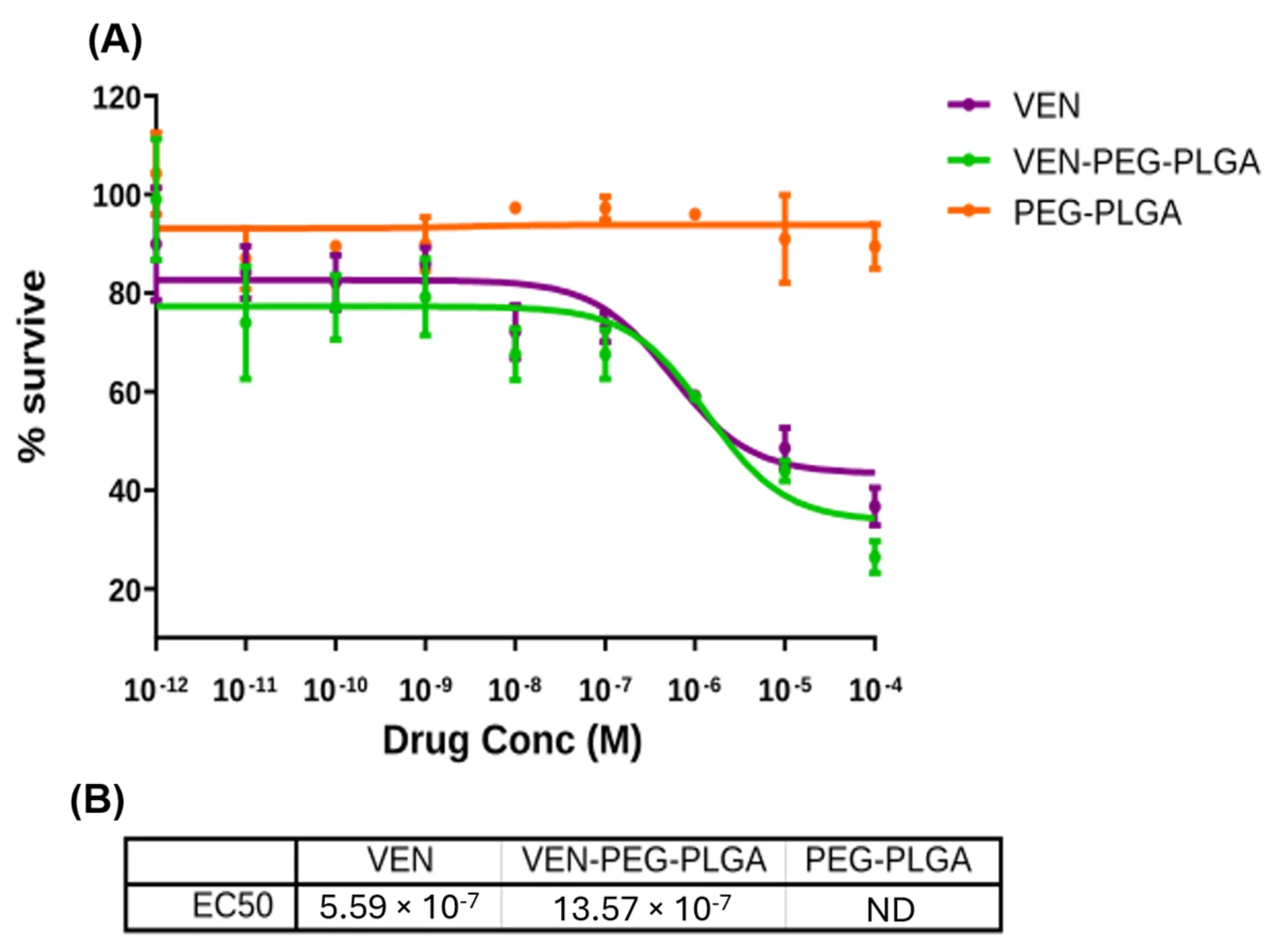
Cytotoxic effects of free venetoclax (VEN), VEN-PEG-PLGA nanoparticles (VEN-PEG-PLGA NPs), and blank PEG-PLGA nanoparticles in THP-1 cells after 48 h of treatment. Cell viability was assessed using the CCK-8 assay. (**A**) Dose–response curves for the different treatment groups. (**B**) EC50 values calculated for free VEN and VEN-PEG-PLGA NPs. ND, not determined. Data are presented as mean ± SD (*n* = 3).

**Figure 4:**
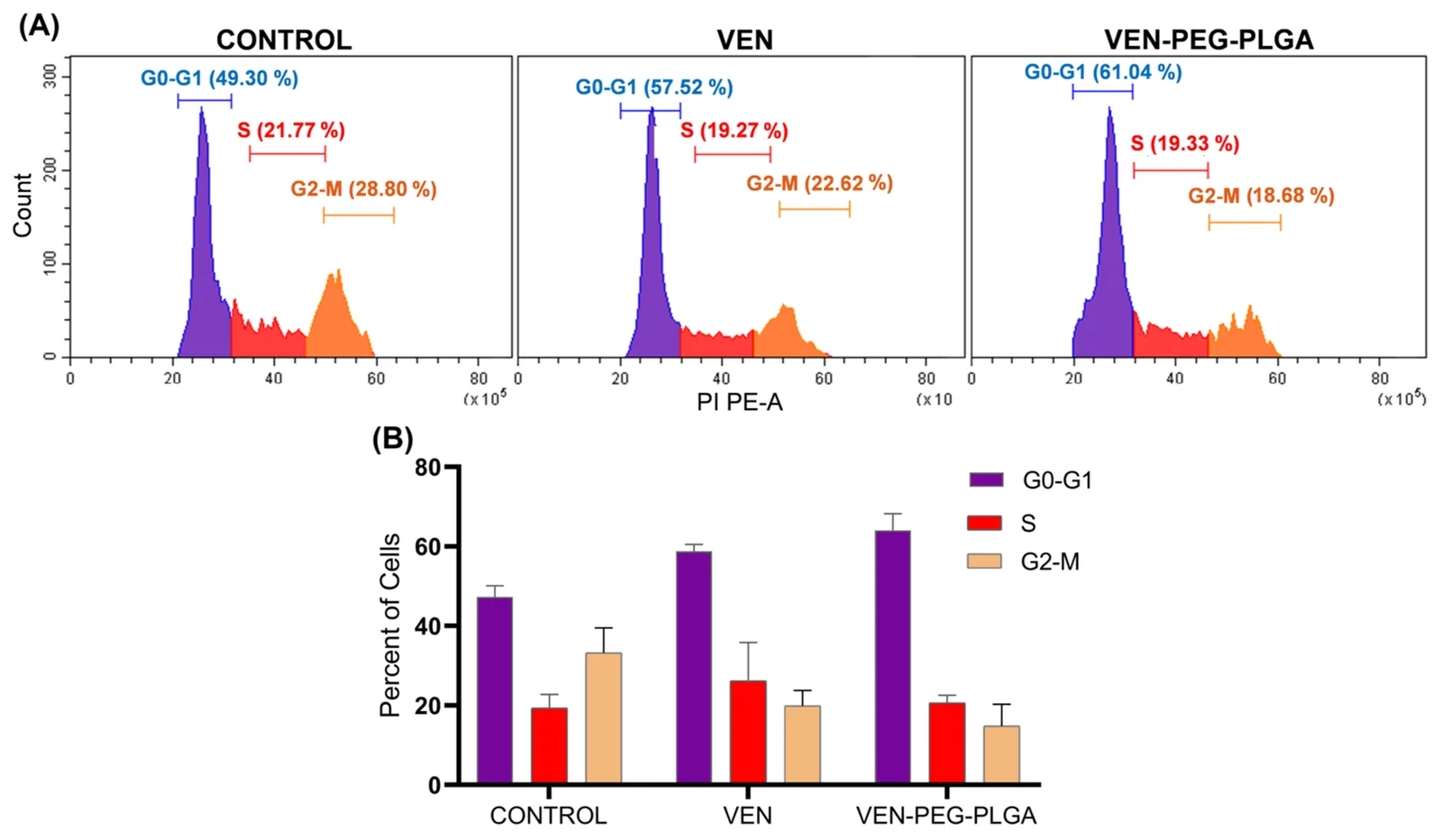
Cell cycle profiles of THP-1 cells treated with free venetoclax (VEN) or VEN-PEG-PLGA nanoparticles (VEN-PEG-PLGA NPs). (**A**) Representative flow cytometric profiles of G0/G1, S, and G2/M phase distributions. (**B**) Quantification of cell cycle populations. Data are mean ± SD (*n* = 3). One-way ANOVA with Tukey’s post hoc test was used (*p* > 0.05).

**Figure 5:**
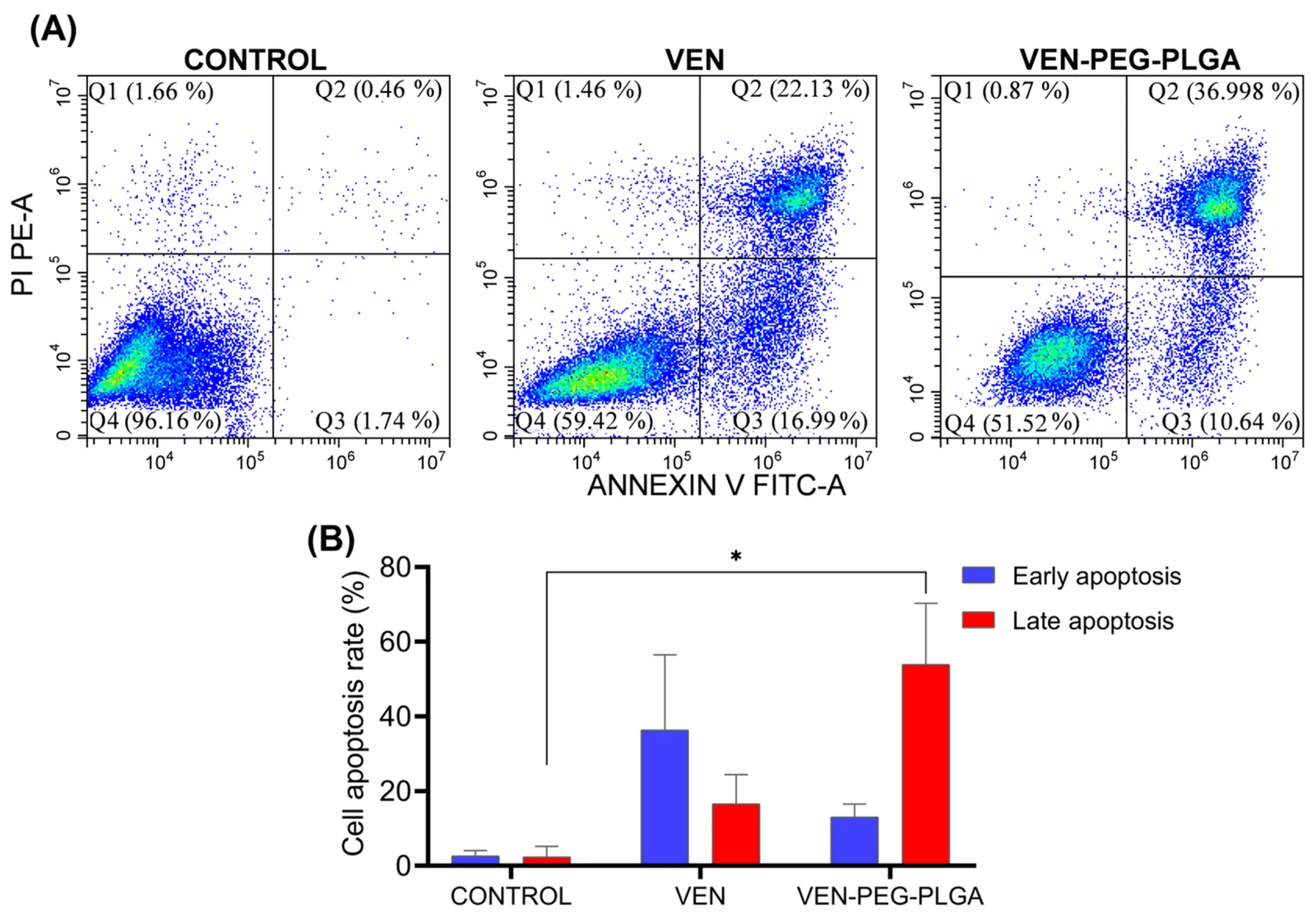
Apoptosis analysis of THP-1 cells treated with free venetoclax (VEN) or VEN-PEG-PLGA nanoparticles (VEN-PEG-PLGA NPs) using Annexin V-FITC/PI flow cytometry. (**A**) Representative plots of viable, early/late apoptotic, and necrotic populations. (**B**) Early and late apoptosis quantified as mean ± SD (*n* = 3). *p < 0.05.

**Figure 6:**
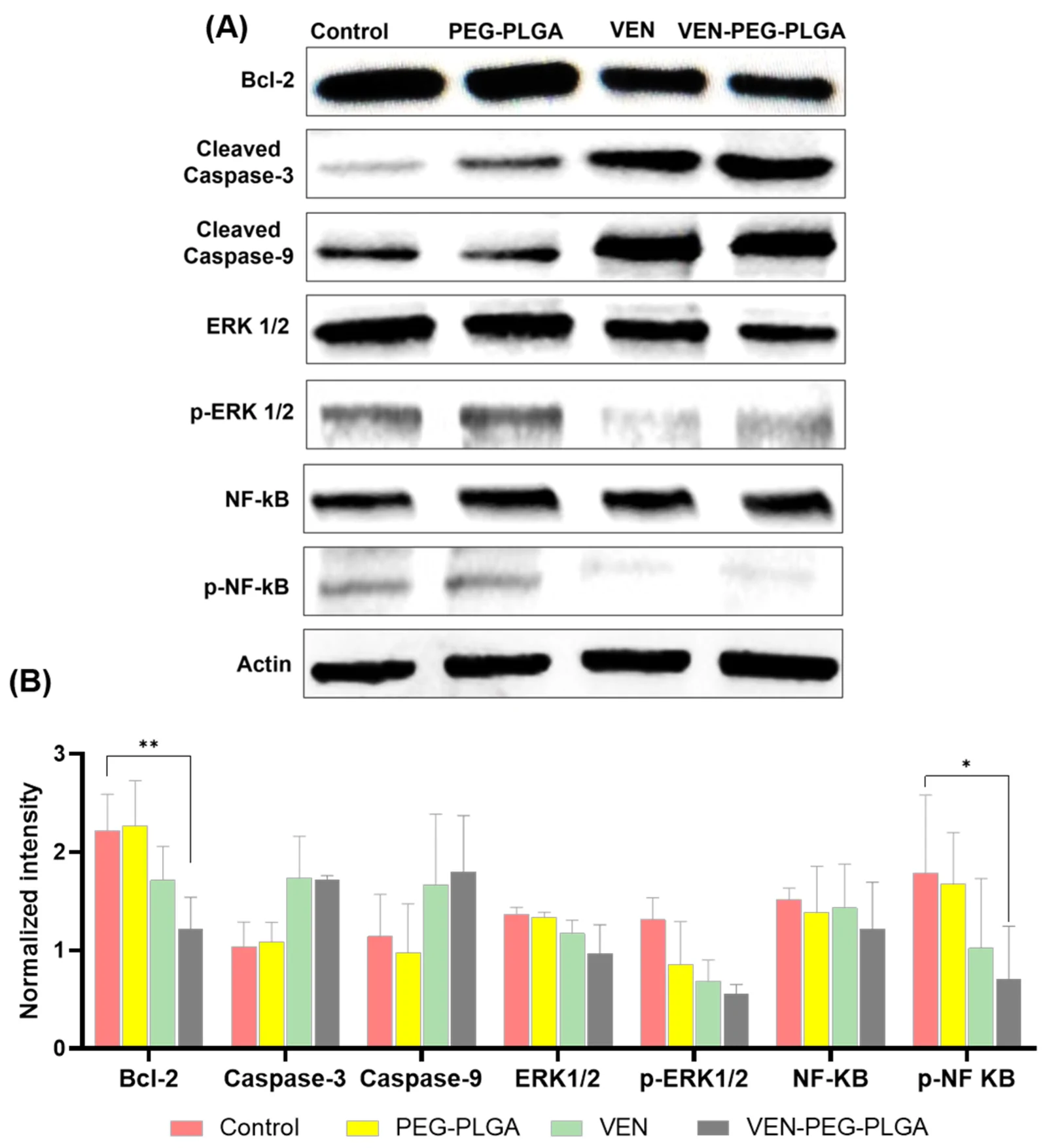
Western blot profiles of THP-1 cells after treatment with VEN, PEG-PLGA NPs, or VEN-PEG-PLGA NPs. (**A**) Bcl-2, cleaved caspase-3/9, ERK1/2, p-ERK1/2, NF-κB, p-NF-κB, and β-actin bands. (**B**) Densitometric analysis normalized to β-actin. Data are mean ± SD (*n* = 3). * *p* < 0.05; ** *p* < 0.01.

## Data Availability

Study data are available upon reasonable request from the corresponding author.

## Abbreviations

The following abbreviations are used in this manuscript:

AML	Acute Myeloid Leukemia
NPs	Nanoparticles
BCL-2	B-cell Lymphoma-2 Protein
DLS	Dynamic Light Scattering
DL%	Drug Loading Percentage
EPR	Enhanced Permeability and Retention Effect
ERK1/2	Extracellular Signal-Regulated Kinase 1/2
GMP	Good Manufacturing Practice
EC50	Half-Maximal Effective Concentration
MAPK	Mitogen-Activated Protein Kinase
MOMP	Mitochondrial Outer Membrane Permeabilization
MTT	3-(4,5-Dimethylthiazol-2-yl)-2,5-Diphenyltetrazolium Bromide
NF-κB	Nuclear Factor Kappa-B
PDI	Polydispersity Index
PEG-PLGA	Polyethylene Glycol-Poly (lactic-co-glycolic acid)
PI	Propidium Iodide
SEM	Scanning Electron Microscopy
VEN-PEG-PLGA NPs	Venetoclax-Loaded PEG-PLGA Nanoparticles
CI	Confidence Interval
LE	Loading Efficiency
LC	Loading Capacity
